# Endoscopic ultrasound criteria to predict the need for intervention in pancreatic necrosis

**DOI:** 10.1186/1471-230X-12-48

**Published:** 2012-05-14

**Authors:** Christian Jürgensen, Alexander Arlt, Frank Neser, Annette Fritscher-Ravens, Ulrich Stölzel, Jochen Hampe

**Affiliations:** 1 Department of Gastroenterology, Charité University Campus Mitte, Berlin, Germany; 2 Interdisciplinary Endoscopy, Department of Medicine I, University Hospital Schleswig-Holstein, Campus Kiel, Kiel, Germany; 3 Department of Medicine II, Klinikum Chemnitz, Chemnitz, Germany

## Abstract

**Background:**

The natural course and treatment strategies for asymptomatic or oligosymptomatic pancreatic necrosis are still poorly defined. The aim of this retrospective study was to establish criteria for the need of intervention in patients with pancreatic necrosis.

**Methods:**

A total of 31 consecutive patients (18 male, median age 58 yrs.) diagnosed with pancreatic necrosis by endoscopic ultrasound, in whom a decision for initial conservative treatment was made, were followed for the need of interventions such as endoscopic or surgical intervention, or death.

**Results:**

After a median follow-up of 243 days, 21 patients remained well without intervention and in 10 patients an endpoint event occurred. In a multivariate logistic regression analysis of the clinical and endosonographic parameters, liquid content was the single independent predictor for intervention (p = 0.0006). The presence of high liquid content in the pancreatic necrosis resulted in a 64% predicted endpoint risk as compared to 2% for solid necrosis.

**Conclusions:**

Pancreatic necrotic cavities with high liquid content are associated with a high risk of complications. Therefore, close clinical monitoring is needed and early elective intervention might be considered in these patients.

## Background

Pancreatic necrosis is a complication of both acute and chronic pancreatitis. Its clinical presentation can range from severe, life-threatening sepsis to oligosymptomatic or even asymptomatic patients. Septic patients with infected pancreatic necrosis on one end of the clinical spectrum of severity are critically ill and require immediate treatment. The range of interventional treatment modalities in these patients includes surgery [1], transgastric endoscopic drainage combined with necrosectomy [2,3], placement of percutaneous drainages and irrigation [4] or combinations of the above modalities. Recent evidence suggests that minimal invasive or endoscopic approaches are superior to open surgery [5-7]. However, even infected necrosis can be managed conservatively in a proportion of patients [8]. Thus, even in this life-threatening condition, the selection of an appropriate treatment modality is an as yet unsolved problem [9].

Given the increased awareness and the improved imaging options of the pancreas such as endoscopic ultrasound, computed tomography and magnetic resonance imaging, more oligosymptomatic or asymptomatic pancreatic lesions including necroses are being identified and represent an additional challenge regarding their adequate management strategy. In contrast to infected necroses, the majority of these patients may be managed conservatively as suggested by recent guidelines [10,11]. However, the technical advances of endoscopic and minimally invasive treatment modalities may lower the threshold of intervention in this patient group. In choosing an interventional strategy, the procedural risk has to be weighed against the likely natural course of the pancreatic necrosis. Data regarding the procedural risk of endoscopic necrosectomy and the recurrence rate in non-infected patients are scarce. Seifert et al. reported a 26% complication rate and a 7.5% mortality rate in 93 patients for endoscopic necrosectomy [7]. This series included 54% infected necroses – therefore, a significant proportion of this morbidity and mortality may be related to the active infection at the time of intervention. On the other hand, persistent or secondary infection of pancreatic necrosis in conservatively treated patients is of particular concern because potentially delayed treatment may lead to intractable sepsis with resultant mortality. The proportion of non-infected necroses that progress to infection depends on the clinical context. In acute pancreatitis necroses are most likely to become infected three to four weeks after the onset of acute pancreatitis [12]. In contrast to studies on acute treatment, data on secondary infection rates after this time frame are rare. Rau et al. reported a mortality rate of 6.2% in non-surgically treated patients with sterile necrosis [13].

The knowledge of factors predicting long-term success of conservative treatment would be critical to make a risk adjusted initial management decision for a newly diagnosed pancreatic necrosis. This study therefore sought to define morphologic endoscopic ultrasound criteria for the prognosis and need of intervention in a series of patients with pancreatic necroses that were initially managed conservatively.

## Methods

### Patients

All consecutive patients meeting the inclusion criteria listed below were included from January 2007 to June 2009 at the regional hospital Chemnitz and from July 2009 to June 2010 at the university hospital Schleswig-Holstein in Kiel. The following criteria were required: i) a diagnosis of pancreatic necrosis based on EUS morphology and ii) an initial decision for conservative management during the hospital stay or at presentation, when seen as outpatient. All patients had a history of acute and/or chronic pancreatitis.

The presence of pancreatic necrosis was defined on the basis of EUS morphology as utilized previously in the literature [14,15]. The morphological criterion for necrosis itself was the presence of solid material with increased or mixed echogenicity. The differential diagnosis to localized pancreatitis was based on the presence of preserved tissue and ductal structures and vascularization, which are both present in the latter case. The morphological differential diagnosis to pancreatic carcinoma was also made on the basis of vascularization, ductal anatomy and the presence of tissue invasion and metastases. Importantly, adenocarcinomas would have presented with a completely different clinical course during the long term follow up of this study. In cases of doubt, EUS fine needle aspiration was performed. When compared with solely solid necrosis, those with additional fluid were identified as structured hyperechogenic material surrounded by fluid and without evidence of blood flow on color Doppler and demarcation by a hypoechogenic wall corresponding to circumferential fibrotic tissue. The EUS diagnosis of pancreatic necrosis was supported by consistent clinical data, EUS-guided fine needle aspiration and/or other imaging modalities (such as computed tomography) if needed.

Patients in severe pain, with conservatively uncontrollable infection, or with symptomatic gastroduodenal compression leading to inability of oral food intake underwent interventions and were not included in this study. Elevated infection parameters such as C-reactive protein levels were tolerated (and recorded) at discharge, if the clinical and laboratory course indicated a progressive recovery without intervention. Four patients were seen as outpatients. The in-hospital treatment of 27 patients consisted of antibiotics in case of suspected infection, enteral nutrition as soon as possible, and discharge without any intervention. Patients were informed to contact either their physician or hospital in case of fever, pain, or other abdominal symptoms. Patients’ demographics such as age, gender, presenting symptoms and comorbidities were recorded. Primary endpoints were death or the need for intervention related to the pancreatic necrosis such as pancreatic surgery, percutaneous drainage, or endoscopic treatment of necrosis. All patients were followed prospectively by telephone calls for primary endpoints or for other details related to pancreas or pancreatic necrosis.

### Scoring of EUS morphology

**Figure 1:**
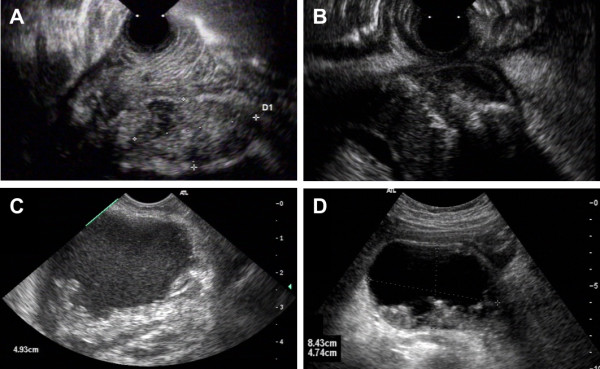
**EUS morphology of pancreatic necrosis.** The figure provides examples of the types of necrosis and the scoring of liquid content and echogenicity as utilized in Table 1 and the analysis. Panel A: Solid pancreatic necrosis (scored as 0 for liquid content and 0 for echogenicity, patient #22 in Table 1); Panel B: Predominantly solid necrosis with echo-free liquid (scored as 1/0 for liquid and echogenicity, patient #2), Panel C: predominantly liquid necrosis with echogenic fluid (scored as 2/1, patient #27) with a diameter of 49 millimeters; Panel D: predominantly liquid necrosis with echo-free fluid in the cavity (scored as 2/0 for liquid and echogenicity, patient #1).

Location and size of the necrotic cavity were documented by EUS. The EUS-morphology of pancreatic necrosis at presentation was classified according to the following parameters: *i)* proportion of solid material (0: completely solid; 1: predominantly solid (>50% of solid material in the cavity) and 2: predominantly fluid) and *ii)* echogenicity of fluid if present (0: echo-free vs. 1: with increased echogenicity). Figure 1 shows some examples of scoring for liquid content and echogenicity of the pancreatic necrotic cavity.

#### EUS examination

EUS examinations were performed by usage of flexible ultrasound video-endoscopes (Pentax EG 3630UR, EG-3870UTK (Pentax Europe GmbH, Hamburg, Germany) or GF-UCT160-AT8 (Olympus Deutschland GmbH, Hamburg, Germany).

#### Statistical analysis

Statistical analysis was performed using SPSS version 18.0 (IBM Corp, Somers, NY 10589, USA) and R (www.r-project.org). Logistic regression with step-wise forward inclusion of variables was performed with SPSS, logistic regression and linear regression with R used the glm() and lm() functions. The function glm() is the standard implementation in R to fit generalized linear models and is used with the “binomial” argument to fit logistic regression models [16,17]. Similarly lm() is used to fit linear models in R [18]. Strobe statement see Additional file 1.

### Ethics

Because of the retrospective observational study design without alterations in the institutional standard of care, no review committee was consulted.

## Results

Thirty-one patients (58% male, median age 58 years) were followed prospectively after the initial decision for conservative treatment for a median of 243 days (range 60 – 922 days). During follow-up, 21 of these patients (68%) remained well without any pancreatic interventions; 20 patients remained symptom-free and one patient reported minor discomfort. Ten patients reached the study endpoint, either requiring intervention or suffering complications of their pancreatic necrosis. Of these, three patients underwent endoscopic necrosectomy, two percutaneous and one endoscopic drainage of their infected pancreatic necrosis, one patient with advanced lung cancer died from sepsis not influenced by endoscopic drainage, one other died from ileus before surgery could be performed, and two had open surgery (one for pancreatic necrosis and one for carcinoma of the pancreatic head). The latter had not been diagnosed 16 months earlier when presenting with focal necrotizing pancreatitis within the pancreatic tail not accompanied by any pancreatic duct dilation. An overview of all patient characteristics and outcomes is given in Table 1.

**Table 1:** Characteristics and endpoints of all 31 patients with inclusion criteria: pancreatic necrosis as diagnosed by EUS + initial conservative treatment

Characteristics at presentation:	
·	Age: median 58 years [range 21 - 82]
·	Gender: male (n = 18); female (n = 13)
·	Pain in 2 patients (6%)
·	C-reactive peptide (CRP): median 12 mg/l [range <0,4 – 107] (not available in 7 patients)
·	Diameter of necrosis: median 44 mm [range 16 - 110]
·	Echogenicity of fluid: increased in 17 patients (54%)
·	Air bubbles in necrotic cavity: 1 patient (3%)
·	Interval to acute pancreatitis or acute attack of chronic pancreatitis: median 119 d [19 – 1460] (not available in 17 patients with chronic pancreatitis or incidental finding of necrosis)
Follow up: Median 243 days [60 – 922]	
Outcome:	
·	without primary endpoint (n = 21)
·	with primary endpoints (n = 10)
	o Necrosectomy (n = 4)
	3 x endoscopic necrosectomy, 1x open surgery
	o Other surgery (n = 1): pancreatic head carcinoma
	o Drainage (n = 4)
	2 x percutaneous drainage; 2 x endosopic drainage (including one patient dying from sepsis)
	o Other death (n = 1): ileus

Upon variable analysis of predictors of patient outcome (i.e. reaching the endpoint), both liquid score (deviance based χ^2^ statistic, p_D_ = 5.75 × 10^-4^) and the diameter of the necrosis (p_D_ = 6.68 × 10^-4^) were significantly associated with outcome. None of the other clinical or demographic parameters such as age, gender, level of C-reactive protein (CRP), pain upon presentation, echogenicity and air content were associated with reaching the endpoint. For seven patients, CRP levels were not available for analysis. The missing values were distributed between the endpoint groups similarly to the overall cohort (five missing in patients without an endpoint score and two in patients that reached the endpoint). The available data do not suggest an impact of CRP on outcome (p_D_ = 0.81). The descriptive statistics and single point p-values are provided in Table 2.

**Table 2:** Single variable analyses of potential predictors of outcome in conservatively treated pancreatic necrosis

	Descriptive statistics		Logistic regression analysis	
Parameter	Endpoint (intervention, death)	No endpoint (uneventful follow-up)	Wald statistic (p-value)	Deviance based statistic (p-value)
Age (median years)	62.5	57	0.558	0.551
Gender (% male)	40%	67%	0.167	0.160
CRP (median mg/dl)	8	19	0.808	0.809
Presence of pain (%)	10%	5%	0.587	0.591
Liquid score (with score: 0/1/2)	0% /20% / 80%	48% /28 %/24%	0.009	5.75 × 10^-4^
Echogenicity (scored as high)	50 %	57 %	0.709	0.710
Presence of air in the necrosis	0%	5%	0.994	0.373
Diameter (median millimeters)	73	37	0.007	6.68 × 10^-4^

CRP, C-reactive peptide.

**Figure 2:**
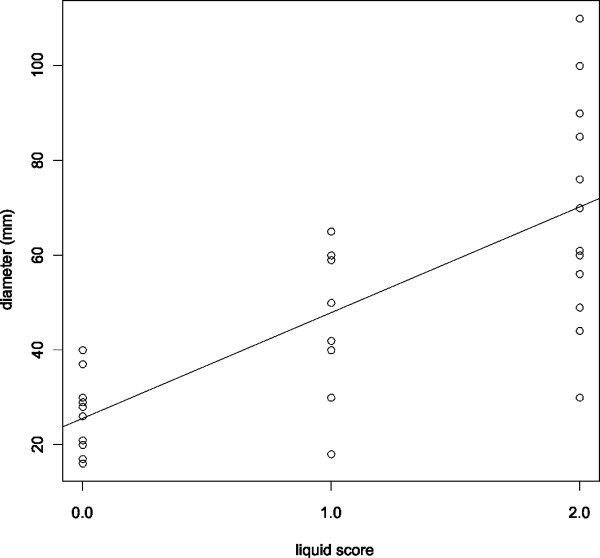
**Liquid score and diameter**. The correlation of the liquid score and diameter of the necrosis is shown in this scatter-plot. The line corresponds to the fitted linear model (F-statistic, p-value: 1.59 × 10^-6^).

The two significant predictors in the single variable analysis, namely the liquid score and the diameter of necrosis were correlated. Figure 2 shows the correlation of the liquid score and the diameter of the pancreatic necrotic cavity as scatterplot and the fitted linear model line (r^2^ = 0.55, F-statistic p = 1.59 × 10^-6^). In order to define the single most relevant parameter for the prediction of outcome, multiple logistic regression analysis using all potential predictor variables in forward likelihood based inclusion was performed. Here, the liquid score was identified as the single independent predictor (Wald statistic p = 0.009, deviance based statistic p_D_ = 5.75 × 10^-4^). Models constructed by inclusion of the liquid score (deviance statistic _D_ = 5.75 × 10^-4^), the diameter of necrosis (p_D_ = 0.16) and an interaction term (p_D_ = 0.99) confirmed these results.

In fact, none of the ten patients without documented fluid within their pancreatic necrosis on EUS needed any intervention during follow-up (median 133 days, range 60–548). On the basis of the logistic regression model, the risk for reaching the endpoint in patients with solid necrosis was estimated at 3%. In contrast, patients with liquid scores of one (low amounts of liquid present) and two (predominantly liquid content) were estimated 18% and 64%, respectively in the logistic regression model. The direct empirical estimates calculated from the data were very similar, namely 62% (8 out of 13) for liquid necrosis, 25% (2 out of 8) for necrosis with low amounts of liquid and 0% (10 out of 10) for solid necrosis.

## Discussion

The armamentarium for interventional and surgical treatment of pancreatic necrosis and cysts has expanded significantly over the last years [2,4-7]. Although the technical feasibility regarding endoscopic necrosectomy is established and the training level of endoscopists is increasing, a clear definition of indications for intervention is still needed, because a significant proportion of patients with pancreatic necrosis can be managed conservatively [8]. On the other hand, an unduly delayed therapy of the pancreatic necrosis could transform an elective procedure in a stable patient into a high risk intervention in the setting of sepsis. Thus, this study aimed to define EUS criteria to guide the threshold of intervention in pancreatic necrosis that does not require immediate intervention because of sepsis, gastric obstruction or other complications.

In this study, the single most relevant predictor for the need of intervention proved to be the liquid content of the necrotic cavity. In the single predictor analysis, the size of the pancreatic necrosis was also significantly associated with outcome, but proved to be inferior to the liquid score on multivariate logistic regression analysis. Both parameters were highly correlated, which corresponds to the clinical reality – i.e. large necrosis cavities tend to have a predominantly liquid content. Biologically, the presence of undrained liquid may correlate with the chance of a secondary infectious event, since the distance from the blood circulation increases the difficulty to control bacteria by the immune system as well as by systemically given antibiotics while providing a medium for rapid spread of infection through the fluid pool. Indeed, such an infectious event led to intervention in 6 of our 10 patients with an endpoint event. Additionally, fluid could be an indicator of chronic minimal leakage fed by an injured pancreatic duct, leading to problems such as pain or increasing size.

Interestingly, increased echogenicity of fluid was not predictive for treatment necessity during follow up. This corresponds to clinical experience in endoscopically treated patients, that an increase of echogenicity is seen both in putrid fluid as well as in clear dark fluid without evidence of infection. Thus, the morphology of the necrosis as defined by liquid content and diameter defines the clinical course in the long term.

The initial decision for patients with EUS documented pancreatic necrosis for conservative treatment is based on lack of clinical indicators for immediate interventions, i.e. pain, uncontrolled infection, inability of oral nutrition, or suspicion for malignancy. In many patients, the decision based on the presence of these indicative symptoms is not clear-cut but based on a spectrum of clinical variables and personal experience. The non-randomized assignment of patients to conservative treatment is a potential weakness of this study. Being aware of this limitation, the long-term clinical follow-up provides a hard clinical outcome measure of the parameters obtained during the initial clinical evaluation. We thus hope, that the morphological parameters provided here, can help guide the therapeutic management resulting in a judicious use of endoscopic necrosectomy in the future.

## Conclusions

Patients with necrotic pancreatic cavities of predominantly liquid content should be monitored closely and may be considered for earlier, elective interventional treatment, because this patient group has a significant risk for severe complications in their clinical course. Conversely, solid necrosis may be relatively safely managed conservatively. Further, preferably randomized studies on this subject are needed.

## Competing interests

The authors declare that they have no competing interests.

## Authors' contributions

CJ, AF and JH participated in conception and design of the study. CJ, AA, and JH participated in drafting of the article. All authors were involved in analysis and interpretation of the study and in provision of study patients. All authors read and approved the final manuscript.

## Pre-publication history

The pre-publication history for this paper can be accessed here:

http://www.biomedcentral.com/1471-230X/12/48/prepub

## Supplementary Material

Additional file 1 **Table S1.** STROBE Statement. Click here for file
